# Fluctuation of Serum Sodium and Its Impact on Short and Long-Term Mortality following Acute Pulmonary Embolism

**DOI:** 10.1371/journal.pone.0061966

**Published:** 2013-04-19

**Authors:** Austin Chin Chwan Ng, Vincent Chow, Andy Sze Chiang Yong, Tommy Chung, Leonard Kritharides

**Affiliations:** Cardiology Department, Concord Hospital, The University of Sydney, Sydney, Australia; Cardiff University, United Kingdom

## Abstract

**Background:**

Baseline hyponatremia predicts acute mortality following pulmonary embolism (PE). The natural history of serum sodium levels after PE and the relevance to acute and long-term mortality after the PE is unknown.

**Methods:**

Clinical details of all patients (n = 1023) admitted to a tertiary institution from 2000–2007 with acute PE were retrieved retrospectively. Serum sodium results from days 1, 3–4, 5–6, and 7 of admission were pre-specified and recorded. We excluded 250 patients without day-1 sodium or had <1 subsequent sodium assessment, leaving 773 patients as the studied cohort. There were 605 patients with normonatremia (sodium≥135 mmol/L throughout admission), 57 with corrected hyponatremia (day-1 sodium<135 mmol/L, then normalized), 54 with acquired hyponatremia and 57 with persistent hyponatremia. Patients’ outcomes were tracked from a state-wide death registry and analyses performed using multivariate-regression modelling.

**Results:**

Mean (±standard deviation) day-1 sodium was 138.2±4.3 mmol/L. Total mortality (mean follow-up 3.6±2.5 years) was 38.8% (in-hospital mortality 3.2%). There was no survival difference between studied (n = 773) and excluded (n = 250) patients. Day-1 sodium (adjusted hazard ratio [aHR] 0.89, 95% confidence interval [CI] 0.83–0.95, *p* = 0.001) predicted in-hospital death. Relative to normonatremia, corrected hyponatremia increased the risk of in-hospital death 3.6-fold (95% CI 1.20–10.9, *p* = 0.02) and persistent hyponatremia increased the risk 5.6-fold (95% CI 2.08–15.0, *p* = 0.001). Patients with either persisting or acquired hyponatremia had worse long-term survival than those who had corrected hyponatremia or had been normonatremic throughout (aHR 1.47, 95% CI 1.06–2.03, *p* = 0.02).

**Conclusion:**

Sodium fluctuations after acute PE predict acute and long-term outcome. Factors mediating the correction of hyponatremia following acute PE warrant further investigation.

## Introduction

Acute pulmonary embolism (PE) is the most severe form of venous thromboembolic disease and the third largest cause of cardiovascular death after coronary artery disease and stroke [1], [2]. Predictors of acute mortality following acute PE include: age>70 years, coexistent malignancy, heart failure, pulmonary disease, systemic hypotension, right ventricular dysfunction, and biomarkers such as cardiac troponins and B-type natriuretic peptide [3]–[6]. The increased mortality risk for survivors of acute PE extends beyond the short-term, with 1-year mortality rates of up to 25% [7], [8], and a 5-year cumulative mortality of over 30% in contemporary cohorts [9].

Hyponatremia is associated with adverse prognosis following acute coronary syndromes [10], patients with chronic heart failure [11], chronic renal failure on hemodialysis [12], and others [13]. Recently, baseline serum hyponatremia was shown to be an independent predictor of 30-day mortality after acute PE [14]. However, rather than being a static biomarker, serum sodium is likely to fluctuate in response to acute illness, fluid balance and other factors such as use of diuretic medications. The natural history of changes to serum sodium following an acute PE is unknown. It is also not known if transient and persistent hyponatremia differentially predict outcome.

The present study reports for the first time the natural history of sodium fluctuation in a large contemporary cohort of patients presenting with acute PE and its impact on acute and long-term outcomes.

## Materials and Methods

### Study Cohort

The main cohort from which the current study is based has been previously reported [9], [15]. In brief, consecutive patients admitted with a principal diagnosis of acute PE between January 2000 and December 2007 were identified retrospectively from a university-affiliated tertiary-referral institution (Concord Hospital, University of Sydney, Australia). The medical records of all identified patients were then reviewed for formal confirmation of diagnosis of acute PE. Confirmed PE was defined according to published guidelines [9], [15], [16]. For those patients who presented on more than one occasion with acute PE during the study period (recurrent PE), only the initial presentation was included. Those patients who were not residents of the local state (New South Wales) during their PE presentation were excluded from the study to minimize incomplete tracking of long-term outcomes.

### Ethics Statement

The study was conducted according to the principles expressed in the Declaration of Helsinki. As the study had a retrospective design and patient data were all de-identified and analyzed anonymously, Concord Hospital Human Research Ethics Committee waived the need for written informed consent and approved the study (CH62/6/2008–009).

### Data Sources

Details of the patients’ admission history including the imaging modality used to diagnose the PE, whether deep vein thrombosis was documented, the admitting physician specialty, length of admission, whether patients were on diuretics on admission, their hemodynamic profiles at admission (heart rate, systolic blood pressure and arterial oxyhemoglobin saturation), blood profiles during admission (serum sodium, creatinine, hemoglobin, and coagulation profiles), and in-hospital outcomes were recorded. Comorbidities including ischemic heart disease, prior coronary artery bypass surgery, heart failure, valvular heart disease, prosthetic heart valves, atrial fibrillation/flutter, peripheral vascular disease, stroke, hypertension, hyperlipidemia, diabetes, current or ex-smoker, types of malignancy, pulmonary disease (asthma and/or emphysema), neurodegenerative disease (dementia and/or Parkinson’s disease), and chronic renal disease coded by diagnosis-related group based on the international classification of diagnosis (ICD-10) were retrieved. In addition, a Charlson Comorbidity Index (CCI) score was assigned to each patient to quantify the comorbidities burden [17], [18].

Serum sodium levels were collected on the following pre-specified time points after admission: day-1 (baseline on admission), days 3 or 4, days 5 or 6, and day-7. If more than one test was performed over the day-range, then the average of the test results was recorded. In order to accurately track sodium fluctuation, we included only patients who had both serum sodium recorded at baseline on admission (day-1) and had at least one subsequent serum sodium assessment during admission (n = 773).

### Study Outcomes

The outcome of the study cohort was tracked using a statewide death registry database. A censored date of 30 June 2008 was pre-determined to allow a minimum follow-up of 6-months. The primary outcome of the study was all-cause mortality. All death certificates were retrieved for review to ascertain the cause of death. Cardiovascular death was defined as death due to PE, acute myocardial infarction, heart failure, stroke, cardiac arrest and cardiac-related causes (when more than one cardiac cause of death was recorded). Non-cardiovascular death included death due to malignancy, sepsis and dementia. Patients with multiple potential causes of death on their death certificates were classified as “undefined” and labeled as non-cardiovascular death for the purposes of the present study. Each cause of death was coded independently by two reviewers (A.N. and L.K.) according to general principles set by the World Health Organization [19]. The reviewers were blinded to patient’s background co-morbid illnesses during coding. Disparities were subsequently resolved by consensus.

### Statistical Analysis

All continuous variables were expressed as mean ± standard deviation, unless otherwise stated, and categorical data given in frequency and percentages. Comparison between groups used unpaired *t* test for continuous variables and *χ* ^2^ tests or Fisher’s exact test for dichotomous variables. Comparison of in-hospital mortality was performed using binary logistic regression analysis. Kaplan-Meier survival methods were used to compare unadjusted long-term survival rates post-discharge. Univariate and multivariate logistic regression analysis was used to assess predictors of in-hospital death, while Cox proportional hazards regression analysis was used to assess predictors of post-discharge death. The univariate predictors that were assessed included age, sex, CCI score (as a continuous variable), comorbidities not included in CCI, whether patients were on diuretics at baseline and laboratory biochemical and hematological parameters. In addition, to adjust for baseline vital signs differences, we used the simplified Pulmonary Embolism Severity Index (sPESI) score, which incorporates age, history of malignancy, heart failure or chronic pulmonary disease, heart rate ≥110 beats per minute, systolic blood pressure <100 mmHg and arterial oxyhemoglobin saturation <90% at admission [20]. The present study first examined the natural history of serum sodium level fluctuation during admission, and then stratified the cohort into four groups [11]: Group 1, patients with normonatremia (serum sodium ≥135 mmol/L) on presentation and throughout admission; Group 2, patients with corrected hyponatremia (initial sodium <135 mmol/L, then normalized during admission); Group 3, patients with acquired hyponatremia after admission (day-1 sodium ≥135 mmol/L, then declined below 135 mmol/L); Group 4, patients with persistent hyponatremia (sodium <135 mmol/L) at baseline and throughout admission. The in-hospital and post-discharge long-term survival outcomes of these patients were compared with group 1 (normonatremic patients) as the reference cohort. In addition, the prognostic significance of baseline serum sodium (day-1 admission) on in-hospital and long-term mortality was assessed. Only univariate variables with *p*<0.10 were included in the multivariate analysis. Analysis was performed using SPSS version 13.0 (SPSS Inc., Chicago, Illinois). A two-tailed probability value <0.05 was considered statistically significant.

## Results

Of 1023 patients admitted with a confirmed diagnosis of PE between 2000 to 2007 [9], 250 patients were excluded from further analysis due to either absence of serum sodium level on day-1 of the index PE admission (40 patients), or because fewer than two serum sodium analyses had been performed during admission (210 patients) (Figure S1). The excluded patients were significantly younger (60.2±17.2 vs. 70.6±15.2years, *p*<0.0001), less likely to be males (39% vs. 46%, *p* = 0.048), had shorter admissions (5.5±4.1 vs. 9.1±6.6days, *p*<0.0001), less likely to receive an echocardiographic study (21% vs. 42%, *p*<0.0001), had lower mean CCI (1.4±2.0 vs. 1.9±2.0, *p* = 0.001) and sPESI (0.6±0.8 vs. 1.1±0.9, *p*<0.0001) scores, better preserved renal function (eGFR 84.5±26.7 vs. 75.2±33.7 ml/min/1.73 m^2^, *p*<0.0001) and higher serum hemoglobin levels (132.9±18.4 vs. 128.7±20.1 g/L, *p* = 0.007) on admission than the included cohort (Table S1). When adjusted for differences in their baseline characteristics, the in-hospital and post-discharge survival of the study group did not differ from that of the excluded group (Figure S2). The final study cohort of 773 patients had a mean follow-up of 3.6±2.5years.

### Baseline Characteristics

**Figure 1:**
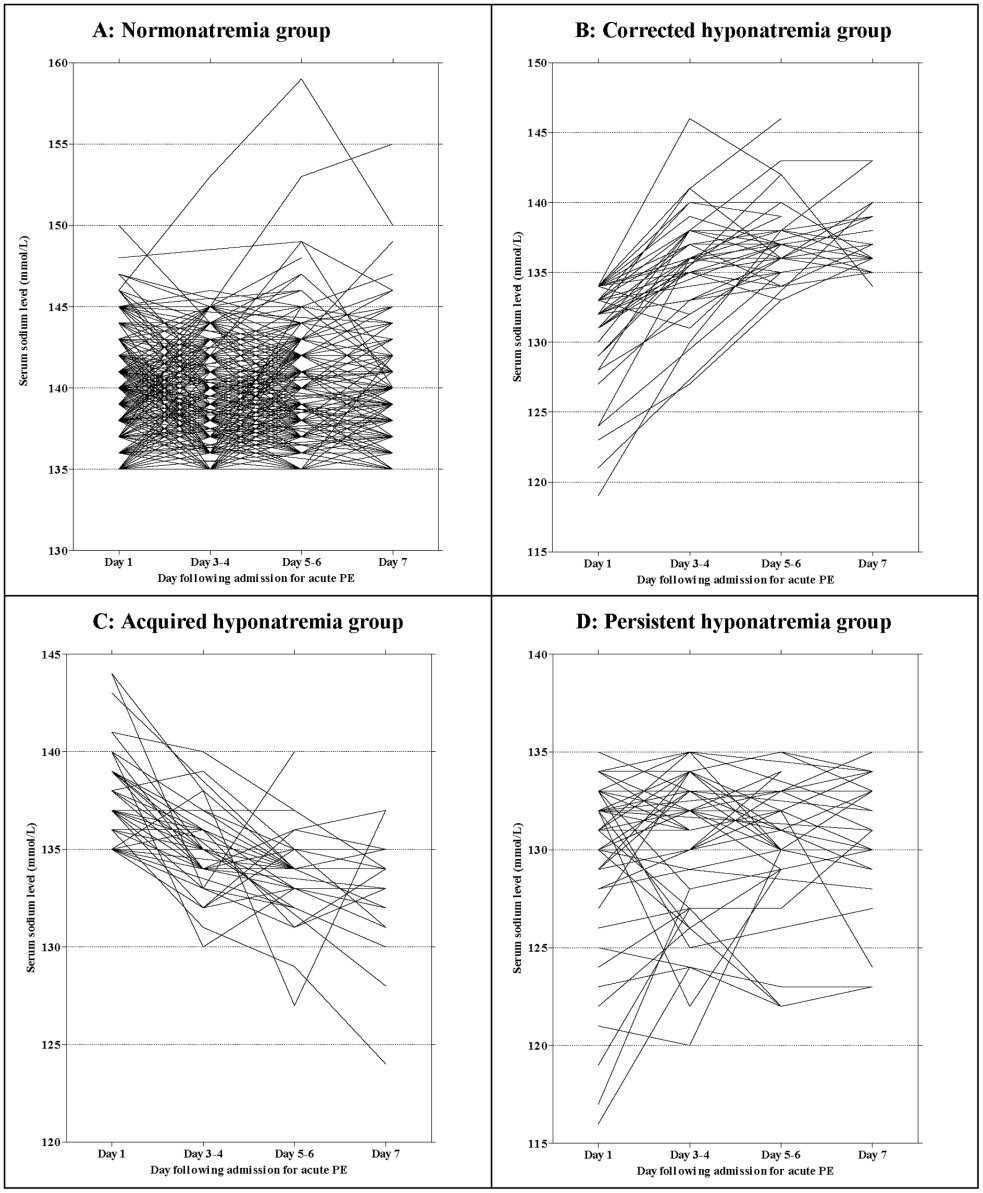
Natural history of serum sodium levels fluctuation during hospital admission for acute PE. The figures show the natural history of the study cohort’s serum sodium levels during the course of their admission for acute PE stratified into the four patterns of sodium fluctuation observed. Each line on the graph represents an individual patient and the time course of that individual’s serum sodium level fluctuations during admission is tracked along the x-axis, which shows the day following admission that individual’s serum sodium was assessed again. Hyponatremia is defined as having a serum sodium level less than 135 mmol/L.

Figure 1 shows the fluctuation of each individual patient’s serum sodium level during admission. Most patients demonstrated serum sodium above 135 mmol/L throughout the admission. Four broad patterns of sodium fluctuation were identified and patients were grouped accordingly: group 1 (normonatremia, n = 605, 78.3%); group 2 (corrected hyponatremia, n = 58, 7.5%); group 3 (acquired hyponatremia, n = 54, 7.0%); and group 4 (persistent hyponatremia, n = 56, 7.2%). A total of 153 (19.8%) patients had a serum sodium less than or equal to 135 mmol/L on day-1 of admission (39 patients had sodium of 135 mmol/L). Patients were categorized into group 2 if sodium corrected to ≥135 mmol/L on any reading after admission and into group 3 if sodium fell to less than 135 mmol/L at any time during the admission. In general, among patients in group 2, those who corrected their initial hyponatremia maintained their sodium ≥135 mmol/L throughout admission although in one subject, the last recorded sodium value fell below 135 mmol/L (Figure 1B).

Table 1 shows the baseline characteristics of the study cohort stratified into different patterns of serum sodium fluctuations during admission. Patients with persistent hyponatremia (group 4) were older and more likely to have underlying ischemic heart disease and heart failure compared to normonatremic patients (group 1). In addition, malignancy was significantly more common in those with corrected (group 2) and persistent (group 4) hyponatremia than in normonatremic patients (group 1). Patients with persistent hyponatremia had more comorbidities, as reflected in the high mean CCI score. Groups 2 and 4 patients (corrected and persistent hyponatremia respectively) scored significantly higher on the sPESI compared to group 1 patients (normonatremia). Mean serum hemoglobin was lowest in those with acquired and persistent hyponatremia (groups 3 and 4 respectively). There were no differences in sex, documented deep vein thrombosis or mean length of hospital stay across the groups.

**Table 1:** Clinical characteristics at baseline.

		Normonatremia	Hyponatremia during admission		
		(sodium≥135mmol/L)	Corrected	Acquired	Persistent
	Study cohort	Group 1	Group 2	Group 3	Group 4
Parameters	N = 773	N = 605	N = 58	N = 54	N = 56
Mean age (±SD) – years	70.6±15.2	69.7±15.5	73.6±15.8	73.3±15.2	74.3±9.6‡
Males – no. (%)	359 (46)	281 (46)	27 (47)	24 (44)	28 (49)
Documented deep vein thrombosis during admission –no. (%)	143 (18)	111 (18)	12 (21)	11 (20)	9 (16)
Admitting physician specialty – no. (%)					
Internal medicine specialties	761 (98.4)	597 (98.7)	55 (96.5)	52 (96.3)	57 (100)
Surgical specialties	12 (1.6)	8 (1.3)	2 (3.5)	2 (3.7)	0 (0)
Length of hospital stay – days					
Mean (±SD)	9.1±6.6	8.9±6.8	9.0±5.9	10.0±5.1	10.9±5.7
Median (25^th^–75^th^ interquartile range)	7 (6–11)	7 (5–10)	8 (6–11)	9 (6–13)	9 (7–13)
Echocardiogram during admission – no. (%)	328 (42)	248 (41)	24 (42)	29 (54)	27 (47)
On diuretic at presentation – no. (%)	180 (23)	131 (22)	12 (21)	21 (39)‡ §	16 (28)
**Haemodynamic profile at admission – mean±SD**					
Heart rate – beats per minute	89±22	89±22	94±25	94±21	86±20
Systolic blood pressure – mmHg	142±26	143±26	136±27	142±26	139±31
Arterial oxyhemoglobin saturation – %	95±4	95±4	94±7‡	95±4	95±5
**Imaging modality**					
Ventilation-perfusion scintigraphy – no. (%)	647 (84)	502 (83)	49 (84)	50 (93)	46 (82)
High probability – no. (%)	576 (75)	449 (74)	44 (76)	43 (80)	40 (71)
Intermediate probability – no. (%)	62 (8)	45 (7)	4 (7)	7 (13)	6 (11)
Computed tomography pulmonary angiogram – no. (%)	204 (26)	158 (26)	15 (26)	14 (26)	17 (30)
Main pulmonary artery – no. (%)	50 (6)	41 (7)	1 (2)	4 (7)	4 (7)
Segmental and sub-segmental – no. (%)	148 (19)	117 (19)	14 (25)	6 (11)	11 (19)
Both imaging modalities used – no.	79 (10)	56 (9)	6 (11)	10 (19)	7 (12)
**Comorbidities** – no. (%) *					
Cardiovascular disease					
Ischaemic heart disease	183 (24)	134 (22)	12 (21)	15 (28)	22 (39)‡ §
Stroke	30 (4)	18 (3)	4 (7)	4 (7)	4 (7)
Heart failure	119 (15)	83 (14)	12 (21)	9 (17)	15 (26)‡
Atrial fibrillation/flutter	137 (18)	97 (16)	15 (26)	10 (19)	15 (26)
Valvular heart disease	17 (2)	15 (2)	1 (2)	1 (2)	0 (0)
Cardiac risk factors					
Hypertension	250 (32)	187 (31)	17 (30)	25 (46)	21 (37)
Hyperlipidemia	107 (14)	82 (14)	7 (12)	6 (11)	12 (21)
Diabetes	126 (14)	94 (16)	9 (16)	10 (19)	13 (23)
Current smoker	59 (8)	50 (8)	3 (5)	3 (6)	3 (5)
Ex-smoker	136 (18)	109 (18)	8 (14)	10 (19)	9 (16)
Malignancy	187 (24)	129 (21)	22 (38)‡	14 (26)	23 (40)‡
Chronic pulmonary disease	108 (14)	81 (13)	9 (16)	9 (17)	9 (16)
Neurodegenerative disease	58 (8)	45 (7)	6 (11)	3 (6)	4 (7)
Chronic renal disease	48 (6)	35 (6)	4 (7)	5 (9)	4 (7)
Charlson comorbidity index score					
Mean score (±SD)	1.9±2.0	1.7±1.9	2.4±2.2	2.2±1.7	3.2±2.5‡
Simplified Pulmonary Embolism Severity Index (sPESI) score					
Mean score (±SD)	1.1±0.9	1.0±0.9	1.5±1.1‡	1.2±1.0	1.4±0.8‡
**Blood profile during admission – mean (±SD)** †					
Serum sodium on admission – mmol/L	138.2±4.3	139.7±2.6	131.6±3.4‡	137.4±2.2‡ §	129.7±4.3‡ § ^‖^
Estimated GFR – ml/min/1.73 m^2^	75.2±33.7	75.3±30.5	72.6±29.1	71.9±58.0	78.9±39.0
Serum hemoglobin – g/L	128.7±20.1	130.8±19.6	123.9±19.5	120.4±18.2‡	119.4±22.0‡
INR at time of admission	1.2±0.5	1.2±0.4	1.3±0.7	1.4±0.7	1.2±0.3
INR at time of hospital discharge	2.3±0.8	2.3±0.7	2.3±0.9	2.5±0.9	2.8±1.0‡ §

Group 1: Normonatremia (initial serum sodium ≥135 mmol/L and stayed normal during admission); Group 2: Corrected hyponatremia (initial serum sodium <135 mmol/L with subsequent normalization during admission, i.e. ≥135 mmol/L); Group 3: Acquired hyponatremia (initial serum sodium ≥135 mmol/L, with subsequent fall during admission to <135 mmol/L); Group 4: Persistent hyponatremia (initial serum sodium <135 mmol/L and stayed <135 mmol/L during admission).

Estimated GFR = 186×([S_CR_/88.4]^−1.154^)×(age)^−0.203^×(0.742 if female), where estimated GFR = estimated glomerular filtration rate (ml/min/1.73 m2), S_CR_ = serum creatinine concentration (µmol/L), and age is expressed in years; INR, international normalized ratio; SD, standard deviation.

* Neurodegenerative disease includes dementia and Parkinson’s disease. Conditions included in the Charlson Comorbidity Index include myocardial infarction, congestive cardiac failure, peripheral vascular disease, cerebrovascular disease, dementia, chronic obstructive pulmonary disease, connective tissue disease, peptic ulcer disease, liver disease (mild vs. moderate to severe), diabetes (with or without organ damage), hemiplegia, moderate to severe renal disease, any tumor (within last 5 years), lymphoma, leukemia, metastatic solid tumor and acquired immunodeficiency syndrome (AIDS). The simplified Pulmonary Embolism Severity Index incorporates age, history of malignancy, cardiac failure or chronic pulmonary disease, heart rate ≥110 beats per minute, systolic blood pressure <100 mmHg and arterial oxyhemoglobin <90% at admission.

† Laboratory parameters were retrieved in 771/773 (99.7%) for estimated GFR; serum hemoglobin in 769/773 (99.5%); INR on admission in 725/773 (93.8%); INR on discharge in 722/773 (93.4%).

‡ *p*<0.05 compared to Group 1.

§ *p*<0.05 compared to Group 2.

‖ *p*<0.05 compared to Group 3.

### Short and Long-Term Outcomes

There were 25 in-hospital deaths (3.2%), with none occurring in patients with acquired (group 3) hyponatremia (Table 2). Compared to normonatremic patients (group 1), there were proportionally more in-hospital deaths in those with corrected (group 2) and persistent (group 4) hyponatremia after acute PE (2.0% vs. 8.6%, hazard ratio [HR] 4.7, 95% confidence interval [CI] 1.6–13.7, *p* = 0.005; and 2.0% vs. 14.3%, HR 8.2, 95% CI 3.2–21.1, *p*<0.0001 respectively).

**Table 2:** Short and long-term outcome post acute PE.

	Study cohort	Group 1	Group 2	Group 3	Group 4
All-cause mortality	N = 773	N = 605	N = 58	N = 54	N = 56
**Short-term – no. (%, 95%CI)**					
In-hospital*	25 (3.2, 2.2–4.7)	12 (2.0, 1.1–3.4)	5 (8.6, 3.8–18.7)†	0 (0)	8 (14.3, 7.5–25.8)†
30-day	35 (4.5, 3.3–6.2)	17 (2.8, 1.8–4.5)	5 (8.6, 3.8–18.7)	1 (1.9, 0.4–9.7)	12 (21.4, 12.7–33.9)
3-month	70 (9.1, 7.2–11.3)	40 (6.6, 4.9–8.9)	6 (10.3, 4.9–20.8)	5 (9.3, 4.1–20.0)	19 (33.9, 22.9–47.1)
6-month	93 (12.0, 9.9–14.5)	56 (9.3, 7.2–11.8)	10 (17.2, 9.7–29.0)	7 (13.0, 6.5–24.5)	20 (35.7, 24.4–48.9)
**Long-term – no. (%, 95%CI)**					
1-year	135 (17.5, 15.0–20.3)	86 (14.2, 11.7–17.2)	13 (22.4, 13.6–34.7)	13 (24.1, 14.7–37.0)	23 (41.1, 29.1–54.2)
3-year	229 (29.6, 26.5–32.9)	152 (25.1, 21.8–28.7)	22 (37.9, 26.5–50.9)	22 (40.7, 28.7–54.1)	33 (58.9, 45.8–70.9)
5-year	267 (34.5, 31.3–38.0)	178 (29.4, 25.9–33.2)	24 (41.4, 29.6–54.3)	28 (51.9, 38.8–64.6)	37 (66.1, 52.9–77.1)
**Total mortality**	300 (38.8, 35.4–42.3)	202 (33.4, 29.7–37.2)	29 (50.0, 37.5–62.5)	31 (57.4, 44.1–69.7)	38 (67.9, 54.7–78.6)

CI indicates confidence interval.

Group 1: Normonatremia (initial serum sodium ≥135 mmol/L and stayed normal during admission); Group 2: Corrected hyponatremia (initial serum sodium <135 mmol/L with subsequent normalization during admission, i.e. ≥135 mmol/L); Group 3: Acquired hyponatremia (initial serum sodium ≥135 mmol/L, with subsequent fall during admission to <135 mmol/L); Group 4: Persistent hyponatremia (initial serum sodium <135 mmol/L and stayed <135 mmol/L during admission).

* Comparison between groups only performed for in-hospital death using binary logistic regression. For post-discharge comparison, see Kaplan-Meier analyses.

† *p*<0.01 compared to Group 1.

**Figure 2:**
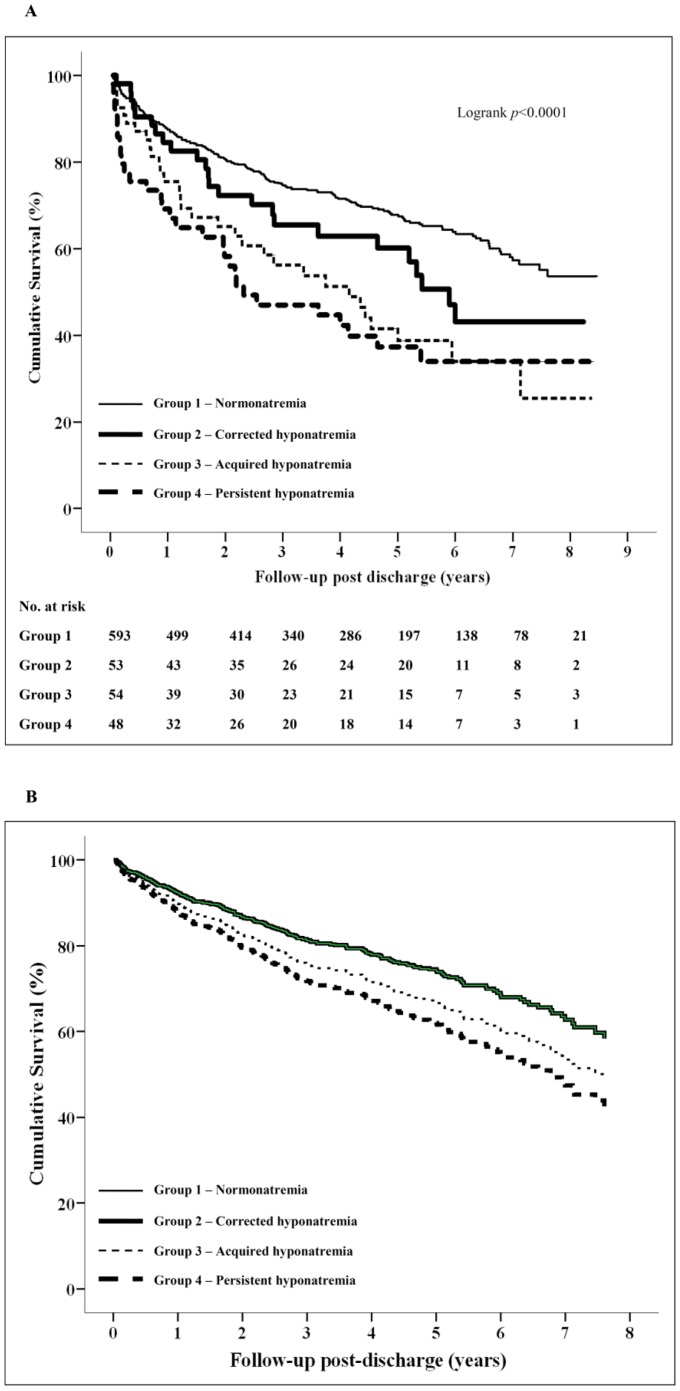
**Figure 2A:** **Kaplan-Meier survival outcome of study cohort post-discharge (stratified by serum sodium group – unadjusted).** The figure shows the unadjusted survival curves of the study cohort stratified into the four patterns of sodium fluctuation observed. Group 1: Normonatremia (initial serum sodium ≥135 mmol/L and stayed normal during admission); Group 2: Corrected hyponatremia (initial serum sodium <135 mmol/L with subsequent normalization during admission, i.e. ≥135 mmol/L); Group 3: Acquired hyponatremia (initial serum sodium ≥135 mmol/L, with subsequent fall during admission to <135 mmol/L); Group 4: Persistent hyponatremia (initial serum sodium <135 mmol/L and stayed <135 mmol/L during admission). **Figure 2B:** **Adjusted Kaplan-Meier survival outcome of study cohort post-discharge (stratified by serum sodium group).** The figure shows the adjusted survival curves of the study cohort stratified into the four patterns of sodium fluctuation observed. Group 1: Normonatremia (initial serum sodium ≥135 mmol/L and stayed normal during admission); Group 2: Corrected hyponatremia (initial serum sodium <135 mmol/L with subsequent normalization during admission, i.e. ≥135 mmol/L); Group 3: Acquired hyponatremia (initial serum sodium ≥135 mmol/L, with subsequent fall during admission to <135 mmol/L); Group 4: Persistent hyponatremia (initial serum sodium <135 mmol/L and stayed <135 mmol/L during admission). The survival curves are adjusted for age (per 1-year), Charlson Comorbidity Index score (per 1-score), whether patient had atrial fibrillation and/or flutter, current smoker status, diuretic use on presentation, the estimated glomerular filtration rate (per 1 ml/min/1.73 m^2^) and serum hemoglobin level on admission. The adjusted survival curve of group 2 patients was identical to that of group 1 patients (the curves superimposed on each other).

Of the 748 patients who survived to discharge, 275 died during follow-up, giving rise to a total mortality of 38.8% (Table 2). Both in-hospital and post-discharge mortality had a significant linear relationship to day-1 (baseline) serum sodium level (*p*<0.005 for trend). Mortality decreased with increasing baseline serum sodium level (Figure S3). Figure 2A shows the unadjusted Kaplan-Meier survival curves of the study cohort post-discharge stratified into the four patterns of serum sodium changes. Compared to normonatremic patients (group 1), those with corrected hyponatremia (group 2) had a non-significantly increased all-cause mortality (32.0% vs. 45.3%, HR 1.4, 95% CI 0.9–2.2, *p* = 0.11). In contrast, patients with acquired hyponatremia (group 3) or persistent hyponatremia (group 4) had significantly worse survival than normonatremic (group 1) patients (32.0% vs. 57.4%, HR 2.1, 95% CI 1.4–3.1, *p*<0.0001; and 32.0% vs. 62.5%, HR 2.5, 95% CI 1.7–3.6, *p*<0.0001 respectively).

Of the total 300 deaths that occurred in-hospital and post-discharge, 41% (n = 122) were cardiovascular-related, and of these pulmonary embolism (n = 32) and acute myocardial infarction (n = 31) were the two main causes (Table S2). Death as a result of sepsis (n = 64) and malignancy (n = 67) accounted for nearly three-quarters of non-cardiovascular causes (n = 178, 59%). No differences in the causes of death were observed across the four groups of patients defined by pattern of serum sodium changes. Only four patients received thrombolytic therapy during admission. None died in-hospital and one patient died of cardiac-related cause post-discharge.

### Serum Sodium as a Predictor of In-Hospital and Post-discharge Mortality Following Acute PE

Table 3 shows the multivariate predictive significance of baseline (day-1) serum sodium level and serum sodium change pattern on in-hospital and post-discharge long-term mortality following acute PE. For in-hospital mortality, there was an 11% decrease in mortality per 1 mmol/L greater serum sodium level measured on day-1 of admission (HR 0.89, 95% CI 0.83–0.95, *p* = 0.001). Compared to normonatremic patients (group 1), those with corrected (group 2) or persistent (group 4) hyponatremia had significantly greater in-hospital mortality (HR 3.62, 95% CI 1.20–10.9, *p* = 0.02; and HR 5.59, 95% CI 2.08–15.0, *p* = 0.001, respectively).

**Table 3:** All-cause mortality and serum sodium fluctuation post acute PE.*

Hyponatremia Variable	In-hospital	*p* value	Post-discharge	*p* value
Baseline serum sodium – per 1 mmol/L increase	0.89 (0.83–0.95)	0.001	0.98 (0.95–1.01)	0.11
Serum sodium change pattern				
Group 1– normonatremia versus:	1.00 *(reference)*	–	1.00 *(reference)*	–
Group 2– corrected hyponatremia	3.62 (1.20–10.9)	0.02	1.00 (0.63–1.59)	1.00
Group 3– acquired hyponatremia†	–	–	1.34 (0.87–2.08)	0.19
Group 4– persistent hyponatremia	5.59 (2.08–15.0)	0.001	1.61 (1.04–2.49)	0.03
Groups 1 & 2 versus Groups 3 & 4	2.17 (0.88–5.30)	0.09	1.47 (1.06–2.03)	0.02

* Unless otherwise indicated, data are presented as adjusted hazard ratio (95% confidence interval). Only univariate variables with *p*<0.10 were included in the multivariate analysis. Multivariate logistic regression analysis was performed for in-hospital death, whilst multivariate Cox proportional hazards regression was performed for post-discharge death analysis. For in-hospital death, the multivariate model was adjusted for age (per 1-year), Charlson Comorbidity Index score (per 1-score) and serum hemoglobin level (per 1 g/L). Diuretic use on presentation was not a univariate predictor of in-hospital death. For post-discharge death, the multivariate model was adjusted for age, Charlson Comorbidity Index score, whether patient had atrial fibrillation and/or flutter, current smoker status, diuretic use on presentation, the estimated glomerular filtration rate (per 1 ml/min/1.73 m^2^) and serum hemoglobin level on admission.

† There were no in-hospital deaths in Group 3 patients.

Baseline day-1 serum sodium was not an independent predictor of long-term mortality (HR 0.98, 95% CI 0.95–1.01, *p* = 0.11). In contrast, patients with acquired (group 3) and persistent (group 4) hyponatremia during admission had poorer long-term survival post-discharge following acute PE compared to normonatremic (group 1) patients (HR 1.34, 95% CI 0.87–2.08, *p* = 0.19; and HR 1.61, 95% CI 1.04–2.49, *p* = 0.03, respectively), whilst long-term mortality did not differ between patients with corrected (group 2) hyponatremia and normonatremic (group 1) patients (HR 1.00, 95% CI 0.63–1.59, *p* = 1.00) (Figure 2B). The hazard ratio was 1.47 (95% CI 1.06–2.03, *p* = 0.02) when patients with acquired and persistent hyponatremia (groups 3 and 4) were compared to those with corrected hyponatremia and normonatremic (groups 1 and 2) patients (Figure S4).

Diuretic use at baseline was found not to be a predictor of in-hospital death, but was a significant predictor of long-term survival post-discharge. Although the use of diuretic medications had some influence on serum sodium, it did not alter the prognostic importance of hyponatremia on long-term outcome (Table 4). Similarly, incorporating the sPESI did not alter the significance of hyponatremia on the study outcomes (Table 4 and Table S3).

**Table 4:** Impact of diuretic use and simplified Pulmonary Embolism Severity Index on serum sodium predicting all-cause mortality post-discharge following acute PE.*

Hyponatremia Variable	Model 1	*p* value	Model 2	*p* value	Model 3	*p* value
Baseline serum sodium – per 1 mmol/L increase	0.97 (0.95–1.00)	0.06	0.98 (0.95–1.01)	0.11	0.98 (0.95–1.01)	0.24
Serum sodium change pattern						
Group 1– normonatremia versus:	1.00 *(reference)*	–	1.00 *(reference)*	–	1.00 *(reference)*	–
Group 2– corrected hyponatremia	0.89 (0.57–1.38)	0.60	1.00 (0.63–1.59)	1.00	0.93 (0.58–1.48)	0.74
Group 3– acquired hyponatremia	1.42 (0.96–2.10)	0.08	1.34 (0.87–2.08)	0.19	1.46 (0.95–2.26)	0.09
Group 4– persistent hyponatremia	1.58 (1.06–2.34)	0.02	1.61 (1.04–2.49)	0.03	1.59 (1.02–2.47)	0.04
Groups 1 & 2 versus Groups 3 & 4	1.52 (1.13–2.04)	0.005	1.47 (1.06–2.03)	0.02	1.54 (1.11–2.14)	0.01

* Unless otherwise indicated, data are presented as adjusted hazard ratio (95% confidence interval). Only univariate variables with *p*<0.10 were included in the multivariate analysis. Only post-discharge analysis was performed as diuretic use on presentation was a significant univariate predictor for only post-discharge death and not for in-hospital death. Multivariate model 1 was adjusted for age (per 1-year), Charlson Comorbidity Index score (per 1-score), whether patient had atrial fibrillation and/or flutter, current smoker status, the estimated glomerular filtration rate (eGFR, per 1 ml/min/1.73 m^2^) and serum hemoglobin level on admission (per 1 g/L). Model 2 added diuretic use on presentation. Model 3 was adjusted for the simplified Pulmonary Embolism Severity Index (incorporates age, history of malignancy, cardiac failure or chronic pulmonary disease, heart rate ≥110 beats/minute, systolic blood pressure <100 mmHg and arterial oxyhemoglobin <90% at admission), atrial fibrillation and/or flutter, current smoker status, eGFR, serum hemoglobin level and diuretic use.

## Discussion

The present study report for the first time the patterns of sodium fluctuation in a large contemporary cohort of patients admitted with an acute PE, with significant differences in in-hospital and long-term survival observed between different patterns of sodium fluctuation during admission. Baseline serum sodium level on day-1 of admission independently predicted in-hospital death but did not predict long-term mortality post-discharge. Patients with acquired or persistent hyponatremia during admission had significantly poorer long-term survival compared to normonatremic patients.

The prevalence of hyponatremia at baseline (≤135 mmol/L) in the current study was 19.8%. This is consistent with the prevalence rate following acute PE of 21.1% reported by Scherz et al [14]. While Scherz et al examined the impact of baseline serum sodium on patient’s outcome by categorizing their cohort into 3 groups, serum sodium <130 vs. 130–135 vs. >135 mmol/L, the present study investigated the prognostic significance of baseline serum sodium as a continuous variable. We showed that with each mmol/L of higher serum sodium level a patient presents with during an acute PE event, there was a concomitant 11% decrease in the risk of in-hospital death. The prognostic impact of baseline serum sodium, on the other hand, did not automatically extend into long-term outcome because of fluctuations after admission. Persistent hyponatremia carried a significant increase in long-term mortality whereas baseline hyponatremia which corrected during admission did not. These results clearly distinguish acute and long-term prognostic implications of variations in serum sodium related to PE.

The two comorbidities most likely to have an influence on baseline serum sodium would be heart failure and chronic renal disease. As a whole group, the prevalence of heart failure and chronic renal disease was low (15% and 6% respectively). Scherz et al reported a similar heart failure prevalence rate (16.5%) in their PE cohort [14]. When stratified into the four sodium change patterns in the current study, patients with corrected and persistent hyponatremia were more likely to have underlying heart failure than normonatremic patients, though this was only significant for the persistent hyponatremic group. In contrast, neither the prevalence of chronic renal disease nor the estimated glomerular filtration rate differed between the four groups of patients. As there could be other diseases influencing serum sodium behavior, we used the CCI to provide a semi-quantitative measure of the total burden of comorbidities [18]. Although the mean CCI score was highest in those showing persistent hyponatremia, multivariate analyses showed that the prediction of in-hospital and long-term outcome by the pattern of sodium fluctuations was independent of comorbidity and other variables. In addition, in-hospital use of diuretic medications also did not affect the prognostic importance of sodium fluctuations on outcome in these patients.

Scherz et al demonstrated the prognostic impact of baseline hyponatremia on 30-day mortality post acute PE was independent of the PESI, a validated prognostic score that includes age, gender, comorbid conditions, and vital signs [14]. In the present study, neither baseline hyponatremia or sodium fluctuations influence on mortality was significantly altered by the simplified PESI [20]. The mechanism underlying this prognostic influence of baseline hyponatremia in acute PE is poorly understood. Hyponatremia has been found to be a marker of advanced right heart failure and poor prognosis in patients with pulmonary arterial hypertension, either as a dichotomous or continuous variable [13]. It is also known that hyponatremia is associated with neurohormonal activation in left heart failure [21]. In the present study, only 42% of patients received an echocardiographic study during their PE admission precluding clear associations with ventricular dysfunction. Although we found the prognostic influence of sodium changes was independent of baseline use of diuretic medications, we cannot exclude the possibility that their serum sodium is likely altered during admission by clinical management such as fluid resuscitation and the initiation/cessation or alteration in the dosing of diuretics. Assessment of neurohormonal changes including catecholamine, renin, angiotensin II, aldosterone, vasopressin and brain natriuretic peptide levels, and careful documentation of the use of diuretics and fluid resuscitation during the management of acute PE may help establish potential pathophysiological link between serum sodium and PE outcome.

The present study demonstrates that the natural history of serum sodium changes during admission for acute PE influences in-hospital outcome and long-term survival. Those presenting with normal serum sodium level that is maintained during admission had the best in-hospital survival. For patients who survived to hospital discharge, those with initial hyponatremia that was corrected during admission had similar adjusted long-term survival to normonatremic patients, while patients who acquired hyponatremia during admission or had persistent hyponatremia had the worst long-term survival. It is possible that the latter patients may have other underlying unrecognized conditions such as hypothyroidism or adrenal insufficiency that left untreated can impact their long-term outcome. In addition, iatrogenic hyponatremia is not uncommon in hospitalized patients through use of fluid resuscitation and diuretics, and failure to maintain normal sodium levels could indicate unrecognized heart failure. Our study cannot distinguish between fluctuations of serum sodium acting as a marker or as a causal influence on outcome. At the very least, based on our data, those patients with hyponatremia on discharge should be considered as warranting careful long-term surveillance. This may include follow-up assessments for potential contributing factors to their persistent hyponatremia such as hypothyroidism, adrenal insufficiency, iatrogenic hyponatremia and unrecognized heart failure. Careful clinical review may declare if these patients later manifest disease which may be amenable to earlier treatment.

The current study limitations included its single-center source of patients and its retrospective design. Our results may not be applicable to patients with small PE who were deemed not to require hospital admission, or to patients with massive PE who died before hospital presentation. In other respects our population is representative of contemporary elderly cohorts with multiple comorbidities and should be clinically relevant to populations outside the tertiary care hospital setting. In addition, the observed acute and long-term mortality of the current cohort is consistent with those reported from registry [4], [22]. As our outcome data were obtained from a statewide death registry, we cannot exclude the possibility that some of the survivors died in other states. However, based on known migration rates, the estimated non-captured deaths during the study period is expected to be at most 0.6% [23]. Our classification of death based on patient’s death certificate followed the World Health Organization guideline [19]. It is possible that some of the PE-related deaths may have been misclassified without formal autopsy. Our overall autopsy rate was only 2.7% (10 out of 300 deaths). This low rate is consistent with a known general trend towards fewer autopsies being performed in recent decade [24], [25]. Our study’s inclusion criteria on serum sodium excluded 24% of patients with PE from analysis. The in-hospital and post-discharge survival of the study group however, did not differ from the excluded group. Although we showed that the prognostic significance of the sodium fluctuations was independent of baseline use of diuretics, future studies should examine the relationship between treatments received by patients after admission for acute PE and their impact on serum sodium. We also do not have accurate information on the duration of anticoagulation therapy in these patients. It is reasonable to expect that almost all of our patients would have received therapy according to national and international guidelines and received between 3–6 months of anticoagulation for a first (non-recurrent) PE [26]. As 90% of deaths in our cohort were not attributable to PE recurrence, our overall conclusions regarding all-cause mortality are unlikely to be influenced by the duration of anticoagulation therapy. This conclusion would be consistent with findings of Schulman et al who found duration of anticoagulation did not impact on long-term outcomes post venous thromboembolism [27].

In summary, patterns of serum sodium fluctuation during acute pulmonary embolism independently predict in-hospital and long-term survival. Factors mediating the correction of hyponatremia following acute PE warrant further investigation.

## Supporting Information

Figure S1 **Derivation of study cohort.**
(DOC)

Figure S2 **Adjusted Kaplan-Meier survival outcome of sodium group versus excluded group.** The thick line represents the final study cohort (sodium group), while the dotted line represents the excluded cohort. The survival curves are adjusted for age (per 1-year), gender, Charlson Comorbidity Index score (per 1-score), estimated GFR (per 1 ml/min/1.73 m^2^) and serum hemoglobin (per 1 g/L). There was no significant difference between the survival curves (adjusted hazard ratio 1.11, 95% CI 0.80–1.54, *p* = 0.52). There was also no difference in in-hospital deaths between the two groups (adjusted hazard ratio 1.45, 95% CI 0.41–5.07, *p* = 0.56). The survival curves also did not differ significantly when adjusted for the simplified Pulmonary Embolism Severity Index score (per 1-score), gender, estimated GFR and serum hemoglobin (adjusted hazard ratio 1.25, 95% CI 0.90–1.73, *p* = 0.18). In-hospital deaths did not differ when adjusted using these variables (adjusted hazard ratio 2.01, 95% CI 0.60–6.82, *p* = 0.26).
(DOC)

Figure S3 Figure S3A: Proportions of in-hospital deaths in relation to Day-1 serum sodium level on admission. The bars show the proportion of in-hospital deaths (in percentage) in each of the serum sodium group. The latter is stratified equally into 9 groups based on patient’s day-1 serum sodium level. The number above each bar represents the total number of patients in each group. Linear trend for in-hospital death was significant with increasing day-1 serum sodium levels (*p*<0.0001). Figure S3B: Proportions of post-discharge deaths in relation to Day-1 serum sodium level on admission. The bars show the proportion of post-discharge deaths (in percentage) in each of the serum sodium group. The latter is stratified equally into 9 groups based on patient’s day-1 serum sodium level. The number above each bar represents the total number of patients in each group. Linear trend for post-discharge death was significant with increasing day-1 serum sodium levels (*p* = 0.003).
(DOC)

Figure S4 **Adjusted Kaplan-Meier survival outcome of study cohort post-discharge (stratified by serum sodium change pattern: Groups 1 and 2 versus 3 and 4).** Group 1: Normonatremia (initial serum sodium ≥135 mmol/L and stayed normal during admission); Group 2: Corrected hyponatremia (initial serum sodium <135 mmol/L with subsequent normalization during admission, i.e. ≥135 mmol/L); Group 3: Acquired hyponatremia (initial serum sodium ≥135 mmol/L, with subsequent fall during admission to <135 mmol/L); Group 4: Persistent hyponatremia (initial serum sodium <135 mmol/L and stayed <135 mmol/L during admission). The survival curves are adjusted for age (per 1-year), Charlson Comorbidity Index score (per 1-score), whether patient had atrial fibrillation and/or flutter, current smoker status, diuretic use on presentation, the estimated glomerular filtration rate (per 1 ml/min/1.73 m^2^) and serum hemoglobin level on admission. The survival curves differed significantly (hazard ratio 1.47, 95% CI 1.06–2.03, *p* = 0.02). The survival curves remained significantly different when adjusted for the simplified Pulmonary Embolism Severity Index score (per 1-score), whether patient had atrial fibrillation and/or flutter, current smoker status, diuretic use on presentation, the estimated glomerular filtration rate (per 1 ml/min/1.73 m^2^) and serum hemoglobin level on admission (hazard ratio 1.54, 95% CI 1.11–2.14, *p* = 0.01).
(DOC)

Table S1 **Clinical parameters of study cohort at baseline.**
(DOC)

Table S2 **Causes of death.**
(DOC)

Table S3 Impact of simplified Pulmonary Embolism Severity Index on serum sodium predicting in-hospital all-cause mortality following acute PE.*
(DOC)

## References

[1] White RH (2003) The epidemiology of venous thromboembolism. Circulation 107: I4–8. 12814979 10.1161/01.CIR.0000078468.11849.66

[2] Giuntini C, Di Ricco G, Marini C, Melillo E, Palla A (1995) Pulmonary embolism: Epidemiology. Chest 107: 3S–9S. 7813326 10.1378/chest.107.1_supplement.3s

[3] Becattini C, Agnelli G (2001) Risk factors for adverse short-term outcome in patients with pulmonary embolism. Thromb Res 103: V239–244. 11567661 10.1016/s0049-3848(01)00291-2

[4] Goldhaber SZ, Visani L, De Rosa M (1999) Acute pulmonary embolism: Clinical outcomes in the international cooperative pulmonary embolism registry (ICOPER). Lancet 353: 1386–1389. 10227218 10.1016/s0140-6736(98)07534-5

[5] Klok FA, Mos IC, Huisman MV (2008) Brain-type natriuretic peptide levels in the prediction of adverse outcome in patients with pulmonary embolism: A systematic review and meta-analysis. Am J Respir Crit Care Med 178: 425–430. 18556626 10.1164/rccm.200803-459OC

[6] Becattini C, Vedovati MC, Agnelli G (2007) Prognostic value of troponins in acute pulmonary embolism: A meta-analysis. Circulation 116: 427–433. 17606843 10.1161/CIRCULATIONAHA.106.680421

[7] Heit JA, Silverstein MD, Mohr DN, Petterson TM, O’Fallon WM, et al (1999) Predictors of survival after deep vein thrombosis and pulmonary embolism: A population-based, cohort study. Arch Intern Med 159: 445–453. 10074952 10.1001/archinte.159.5.445

[8] Spencer FA, Gore JM, Lessard D, Douketis JD, Emery C, et al (2008) Patient outcomes after deep vein thrombosis and pulmonary embolism: The Worcester venous thromboembolism study. Arch Intern Med 168: 425–430. 18299499 10.1001/archinternmed.2007.69 PMC2762782

[9] Ng AC, Chung T, Yong AS, Wong HS, Chow V, et al (2011) Long-term cardiovascular and noncardiovascular mortality of 1023 patients with confirmed acute pulmonary embolism. Circ Cardiovasc Qual Outcomes 4: 122–128. 21098781 10.1161/CIRCOUTCOMES.110.958397

[10] Goldberg A, Hammerman H, Petcherski S, Nassar M, Zdorovyak A, et al (2006) Hyponatremia and long-term mortality in survivors of acute ST-elevation myocardial infarction. Arch Intern Med 166: 781–786. 16606816 10.1001/archinte.166.7.781

[11] Gheorghiade M, Rossi JS, Cotts W, Shin DD, Hellkamp AS, et al (2007) Characterization and prognostic value of persistent hyponatremia in patients with severe heart failure in the ESCAPE Trial. Arch Intern Med 167: 1998–2005. 17923601 10.1001/archinte.167.18.1998

[12] Waikar SS, Curhan GC, Brunelli SM (2011) Mortality associated with low serum sodium concentration in maintenance hemodialysis. Am J Med 124: 77–84. 21187188 10.1016/j.amjmed.2010.07.029 PMC3040578

[13] Forfia PR, Mathai SC, Fisher MR, Housten-Harris T, Hemnes AR, et al (2008) Hyponatremia predicts right heart failure and poor survival in pulmonary arterial hypertension. Am J Respir Crit Care Med 177: 1364–1369. 18356560 10.1164/rccm.200712-1876OC PMC2427057

[14] Scherz N, Labarere J, Mean M, Ibrahim SA, Fine MJ, et al (2010) Prognostic importance of hyponatremia in patients with acute pulmonary embolism. Am J Respir Crit Care Med 182: 1178–1183. 20595225 10.1164/rccm.201003-0481OC PMC3001260

[15] Ng AC, Yong AS, Chow V, Chung T, Freedman SB, et al.. (2011) Cardiac troponin-T and the prediction of acute and long-term mortality after acute pulmonary embolism. Int J Cardiol Aug 22 [Epub ahead of print]. 10.1016/j.ijcard.2011.07.107 21864916

[16] Torbicki A, Perrier A, Konstantinides S, Agnelli G, Galie N, et al (2008) Guidelines on the diagnosis and management of acute pulmonary embolism: The task force for the diagnosis and management of acute pulmonary embolism of the European Society of Cardiology (ESC). Eur Heart J 29: 2276–2315. 18757870 10.1093/eurheartj/ehn310

[17] Charlson ME, Pompei P, Ales KL, MacKenzie CR (1987) A new method of classifying prognostic comorbidity in longitudinal studies: Development and validation. J Chronic Dis 40: 373–383. 3558716 10.1016/0021-9681(87)90171-8

[18] Sundararajan V, Henderson T, Perry C, Muggivan A, Quan H, et al (2004) New ICD-10 version of the Charlson comorbidity index predicted in-hospital mortality. J Clin Epidemiol 57: 1288–1294. 15617955 10.1016/j.jclinepi.2004.03.012

[19] National center for health statistics (2008) Instructions for classifying the underlying cause-of-death, ICD-10. 1–259. Available: http://www.cdc.gov/nchs/about/major/dvs/im.htm. Accessed 2009 Jun 10.

[20] Jimenez D, Aujesky D, Moores L, Gomez V, Lobo JL, et al (2010) Simplification of the Pulmonary Embolism Severity Index for prognostication in patients with acute symptomatic pulmonary embolism. Arch Intern Med 170: 1383–1389. 20696966 10.1001/archinternmed.2010.199

[21] Haley H, Ploth DW (2010) Dyshomeostasis of serum sodium concentration in congestive heart failure. Am J Med Sci 340: 42–47. 20610972 10.1097/MAJ.0b013e3181e5939e

[22] Lobo JL, Zorrilla V, Aizpuru F, Uresandi F, Garcia-Bragado F, et al (2006) Clinical syndromes and clinical outcome in patients with pulmonary embolism: Findings from the RIETE registry. Chest 130: 1817–1822. 17167002 10.1378/chest.130.6.1817

[23] Australian Bureau of Statistics (2007) Available: http://www.abs.gov.au.Accessed 2009 Mar 2.

[24] Royal College of Pathologists of Australasia Autopsy Working Party (2004) The decline of the hospital autopsy: A safety and quality issue for healthcare in Australia. Med J Aust 180: 281–285. 15012566 10.5694/j.1326-5377.2004.tb05926.x

[25] Shojania KG, Burton EC (2008) The vanishing nonforensic autopsy. N Engl J Med 358: 873–875. 18305264 10.1056/NEJMp0707996

[26] Gallus AS, Baker RI, Chong BH, Ockelford PA, Street AM (2000) Consensus guidelines for warfarin therapy. Recommendations from the Australasian Society of Thrombosis and Haemostasis. Med J Aust 172: 600–605. 10914107

[27] Schulman S, Lindmarker P, Holmstrom M, Larfars G, Carlsson A, et al (2006) Post-thrombotic syndrome, recurrence, and death 10 years after the first episode of venous thromboembolism treated with warfarin for 6 weeks or 6 months. J Thromb Haemost 4: 734–742. 16634738 10.1111/j.1538-7836.2006.01795.x

