# Supplementary material for: Fluctuation of Serum Sodium and Its Impact on Short and Long-Term Mortality following Acute Pulmonary Embolism
Source: PLoS One. 2013 Apr 19;8(4):e61966. doi: 10.1371/journal.pone.0061966 (PMC3631139; doi:10.1371/journal.pone.0061966)
Supplement: Figure S1 — Derivation of study cohort. (DOC) [file pone.0061966.s001.doc]

**Online-only Figure S1. Derivation of study cohort.**

| |  | | | | --- | --- | --- | |  | | | | 1023 presentations of confirmed acute PE during study period (2000-2007) | | | |  | | | |  |  |  | |  | | | | Excluded 40 patients without day 1 serum sodium analysis | | | |  | | | |  |  |  | |  | | | | Potential study cohort: 983 patients | | | |  | | | |  |  |  | |  | | | | Excluded 210 patients with <2 serum sodium analyses during index PE admission | | | |  | | | |  |  |  | |  | | | | **Final study cohort: 773 patients** | | | |  | | | |  | | | |
| --- | --- | --- | --- | --- | --- | --- | --- | --- | --- | --- | --- | --- | --- | --- | --- | --- | --- | --- | --- | --- | --- | --- | --- | --- | --- | --- | --- | --- | --- | --- | --- | --- | --- | --- | --- | --- | --- | --- | --- | --- | --- | --- | --- | --- | --- | --- | --- | --- | --- | --- | --- | --- | --- | --- | --- | --- | --- | --- | --- | --- | --- | --- | --- |
