# Supplementary material for: Fluctuation of Serum Sodium and Its Impact on Short and Long-Term Mortality following Acute Pulmonary Embolism
Source: PLoS One. 2013 Apr 19;8(4):e61966. doi: 10.1371/journal.pone.0061966 (PMC3631139; doi:10.1371/journal.pone.0061966)
Supplement: Figure S2 — Adjusted Kaplan-Meier survival outcome of sodium group versus excluded group. The thick line represents the final study cohort (sodium group), while the dotted line represents the excluded cohort. The survival curves are adjusted for age (per 1-year), gender, Charlson Comorbidity Index score (per 1-score), estimated GFR (per 1 ml/min/1.73 m2) and serum hemoglobin (per 1 g/L). There was no significant difference between the survival curves (adjusted hazard ratio 1.11, 95% CI 0.80–1.54, p = 0.52). There was also no difference in in-hospital deaths between the two groups (adjusted hazard ratio 1.45, 95% CI 0.41–5.07, p = 0.56). The survival curves also did not differ significantly when adjusted for the simplified Pulmonary Embolism Severity Index score (per 1-score), gender, estimated GFR and serum hemoglobin (adjusted hazard ratio 1.25, 95% CI 0.90–1.73, p = 0.18). In-hospital deaths did not differ when adjusted using these variables (adjusted hazard ratio 2.01, 95% CI 0.60–6.82, p = 0.26). (DOC) [file pone.0061966.s002.doc]

**Online-only Figure S2. Adjusted Kaplan-Meier survival outcome of sodium group versus excluded group.**

| __  **Study Group**  **Excluded Group** |
| --- |
