# Supplementary material for: Fluctuation of Serum Sodium and Its Impact on Short and Long-Term Mortality following Acute Pulmonary Embolism
Source: PLoS One. 2013 Apr 19;8(4):e61966. doi: 10.1371/journal.pone.0061966 (PMC3631139; doi:10.1371/journal.pone.0061966)
Supplement: Figure S3 — A: Proportions of in-hospital deaths in relation to Day-1 serum sodium level on admission. The bars show the proportion of in-hospital deaths (in percentage) in each of the serum sodium group. The latter is stratified equally into 9 groups based on patient’s day-1 serum sodium level. The number above each bar represents the total number of patients in each group. Linear trend for in-hospital death was significant with increasing day-1 serum sodium levels (p<0.0001). Figure S3B: Proportions of post-discharge deaths in relation to Day-1 serum sodium level on admission. The bars show the proportion of post-discharge deaths (in percentage) in each of the serum sodium group. The latter is stratified equally into 9 groups based on patient’s day-1 serum sodium level. The number above each bar represents the total number of patients in each group. Linear trend for post-discharge death was significant with increasing day-1 serum sodium levels (p = 0.003). (DOC) [file pone.0061966.s003.doc]

**Online-only Figure S3A. Proportions of in-hospital deaths in relation to Day-1 serum sodium level on admission.**

**Online-only Figure S3B. Proportions of post-discharge deaths in relation to Day-1 serum sodium level on admission.**
