# Supplementary material for: Fluctuation of Serum Sodium and Its Impact on Short and Long-Term Mortality following Acute Pulmonary Embolism
Source: PLoS One. 2013 Apr 19;8(4):e61966. doi: 10.1371/journal.pone.0061966 (PMC3631139; doi:10.1371/journal.pone.0061966)
Supplement: Figure S4 — Adjusted Kaplan-Meier survival outcome of study cohort post-discharge (stratified by serum sodium change pattern: Groups 1 and 2 versus 3 and 4). Group 1: Normonatremia (initial serum sodium ≥135 mmol/L and stayed normal during admission); Group 2: Corrected hyponatremia (initial serum sodium <135 mmol/L with subsequent normalization during admission, i.e. ≥135 mmol/L); Group 3: Acquired hyponatremia (initial serum sodium ≥135 mmol/L, with subsequent fall during admission to <135 mmol/L); Group 4: Persistent hyponatremia (initial serum sodium <135 mmol/L and stayed <135 mmol/L during admission). The survival curves are adjusted for age (per 1-year), Charlson Comorbidity Index score (per 1-score), whether patient had atrial fibrillation and/or flutter, current smoker status, diuretic use on presentation, the estimated glomerular filtration rate (per 1 ml/min/1.73 m2) and serum hemoglobin level on admission. The survival curves differed significantly (hazard ratio 1.47, 95% CI 1.06–2.03, p = 0.02). The survival curves remained significantly different when adjusted for the simplified Pulmonary Embolism Severity Index score (per 1-score), whether patient had atrial fibrillation and/or flutter, current smoker status, diuretic use on presentation, the estimated glomerular filtration rate (per 1 ml/min/1.73 m2) and serum hemoglobin level on admission (hazard ratio 1.54, 95% CI 1.11–2.14, p = 0.01). (DOC) [file pone.0061966.s004.doc]

**Online-only Figure S4. Adjusted Kaplan-Meier survival outcome of study cohort post-discharge (stratified by serum sodium change pattern: Groups 1 and 2 versus 3 and 4).**

| __  **Group 1 & 2 – Normonatremia/Corrected hyponatremia**  **Group 3 & 4 – Acquired/Persistent hyponatremia** |
| --- |
