# Supplementary material for: Fluctuation of Serum Sodium and Its Impact on Short and Long-Term Mortality following Acute Pulmonary Embolism
Source: PLoS One. 2013 Apr 19;8(4):e61966. doi: 10.1371/journal.pone.0061966 (PMC3631139; doi:10.1371/journal.pone.0061966)
Supplement: Table S1 — Clinical parameters of study cohort at baseline. (DOC) [file pone.0061966.s005.doc]

| **Online-only Table S1.** Clinical parameters of study cohort at baseline. | | | | |
| --- | --- | --- | --- | --- |
|  | **Confirmed PE cohort** | **Sodium Group *** | **Excluded Group ^†^** | ***p* value** |
| **Parameters** | **N=1023** | **N=773** | **N=250** |  |
| Mean age (±SD) – years | 68.1±16.3 | 70.6±15.2 | 60.2±17.2 | <0.0001 |
| Males – no. (%) | 457 (45) | 359 (46) | 98 (39) | 0.048 |
| Documented deep vein thrombosis during admission – no. (%) | 184 (18) | 143 (18) | 41 (16) | 0.51 |
| Admitting physician specialty – no. (%) |  |  |  |  |
| Internal medicine specialties | 999 (97.1) | 761 (98.4) | 238 (95.2) | 0.007 |
| Surgical specialties | 19 (1.9) | 12 (1.6) | 7 (2.8) | 0.28 |
| Emergency department | 5 (0.5) | 0 (0) | 5 (2.0) | 0.001 |
| Length of hospital stay – days |  |  |  |  |
| Mean (±SD) | 8.3±6.3 | 9.1±6.6 | 5.5±4.1 | <0.0001 |
| Median (25^th^-75^th^ interquartile range) | 7 (5 – 10) | 7 (6 – 11) | 5 (3 – 7) | - |
| Echocardiogram during admission – no. (%) | 381 (37) | 328 (42) | 53 (21) | <0.0001 |
| **Haemodynamic profile at admission – mean±SD** |  |  |  |  |
| Heart rate – beats per minute | 88±21 | 89±22 | 84±18 | <0.0001 |
| Systolic blood pressure – mmHg | 141±25 | 142±26 | 136±20 | <0.0001 |
| Arterial oxyhemoglobin saturation – % | 95±4 | 95±4 | 96±2 | <0.0001 |
| **Imaging modality** |  |  |  |  |
| Ventilation-perfusion scintigraphy – no. (%) | 860 (84) | 647 (84) | 213 (85) | 0.62 |
| High probability – no. (%) | 758 (74) | 576 (75) | 182 (73) | 0.62 |
| Intermediate probability – no. (%) | 90 (9) | 62 (8) | 28 (11) | 0.13 |
| Computed tomography pulmonary angiogram – no. (%) | 257 (25) | 204 (26) | 53 (21) | 0.11 |
| Main pulmonary artery – no. (%) | 58 (6) | 50 (6) | 8 (3) | 0.06 |
| Segmental and sub-segmental – no. (%) | 186 (18) | 148 (19) | 38 (15) | 0.19 |
| Both imaging modalities used – no. | 102 (10) | 79 (10) | 23 (9) | 0.72 |
| **Comorbidities** – no. (%) ^‡^ |  |  |  |  |
| Cardiovascular disease |  |  |  |  |
| Ischaemic heart disease | 210 (21) | 183 (24) | 27 (11) | <0.0001 |
| Stroke | 33 (3) | 30 (4) | 3 (1) | 0.04 |
| Heart failure | 133 (13) | 119 (15) | 14 (6) | <0.0001 |
| Atrial fibrillation/flutter | 157 (15) | 137 (18) | 20 (8) | <0.0001 |
| Valvular heart disease | 23 (2) | 17 (2) | 6 (2) | 0.81 |
| Cardiac risk factors |  |  |  |  |
| Hypertension | 316 (31) | 250 (32) | 66 (26) | 0.08 |
| Hyperlipidemia | 142 (14) | 107 (14) | 35 (14) | 1.00 |
| Diabetes | 158 (15) | 126 (14) | 32 (13) | 0.19 |
| Current smoker | 86 (8) | 59 (8) | 27 (11) | 0.12 |
| Ex-smoker | 177 (17) | 136 (18) | 41 (16) | 0.70 |
| Malignancy | 229 (22) | 187 (24) | 42 (17) | 0.02 |
| Chronic pulmonary disease | 144 (14) | 108 (14) | 36 (14) | 0.92 |
| Neurodegenerative disease | 66 (6) | 58 (8) | 8 (3) | 0.02 |
| Chronic renal disease | 59 (6) | 48 (6) | 11 (4) | 0.35 |
| Charlson comorbidity index score |  |  |  |  |
| Mean score (±SD) | 1.8±2.0 | 1.9±2.0 | 1.4±2.0 | 0.001 |
| Simplified Pulmonary Embolism Severity Index (sPESI) score |  |  |  |  |
| Mean score (±SD) | 0.9±0.9 | 1.1±0.9 | 0.6±0.8 | <0.0001 |
| **Blood profile during admission – mean (±SD)** |  |  |  |  |
| Estimated GFR – ml/min/1.73m^2^ | 77.2±32.5 | 75.2±33.7 | 84.5±26.7 | <0.0001 |
| Serum hemoglobin – g/L | 129.6±19.8 | 128.7±20.1 | 132.9±18.4 | 0.007 |
| INR at time of admission | 1.2±0.5 | 1.2±0.5 | 1.3±0.6 | 0.19 |
| INR at time of hospital discharge | 2.3±0.8 | 2.3±0.8 | 2.2±0.8 | 0.006 |
|  |  |  |  |  |
| Estimated GFR = 186 x ([S_CR_/88.4]^-1.154^) x (age)^-0.203^ x (0.742 if female), where estimated GFR = estimated glomerular filtration rate (ml/min/1.73m2), S_CR_ = serum creatinine concentration (µmol/L), and age is expressed in years; INR, international normalized ratio; SD, standard deviation.   - The Sodium Group were patients with day-1 serum sodium and had subsequent sodium analyses during their admission for acute PE. Laboratory parameters were retrieved in 771/773 (99.7%) for estimated GFR; serum hemoglobin in 769/773 (99.5%); INR on admission in 725/773 (93.8%); INR on discharge in 722/773 (93.4%). - The Excluded Group were patients who either did not have serum sodium analysed on day-1 of their PE admission or had less than two serum sodium analyses performed during their admission.  Neurodegenerative disease includes dementia and Parkinson’s disease. Conditions included in the Charlson Comorbidity Index include myocardial infarction, congestive cardiac failure, peripheral vascular disease, cerebrovascular disease, dementia, chronic obstructive pulmonary disease, connective tissue disease, peptic ulcer disease, liver disease (mild vs. moderate to severe), diabetes (with or without organ damage), hemiplegia, moderate to severe renal disease, any tumor (within last 5 years), lymphoma, leukemia, metastatic solid tumor and acquired immunodeficiency syndrome (AIDS). The simplified Pulmonary Embolism Severity Index incorporates age, history of malignancy, cardiac failure or chronic pulmonary disease, heart rate ≥110 beats per minute, systolic blood pressure <100mmHg and arterial oxyhemoglobin <90% at admission. | | | | |
