# Supplementary material for: Fluctuation of Serum Sodium and Its Impact on Short and Long-Term Mortality following Acute Pulmonary Embolism
Source: PLoS One. 2013 Apr 19;8(4):e61966. doi: 10.1371/journal.pone.0061966 (PMC3631139; doi:10.1371/journal.pone.0061966)
Supplement: Table S2 — Causes of death. (DOC) [file pone.0061966.s006.doc]

| **Online-only Table S2.** Causes of death. | | | | | |
| --- | --- | --- | --- | --- | --- |
|  | **Study cohort** | **Group 1** | **Group 2** | **Group 3** | **Group 4** |
|  | **N=300** | **N=202** | **N=29** | **N=31** | **N=38** |
| **Causes of death – no. (%)** * |  |  |  |  |  |
| Cardiovascular causes | 122 (40.7) | 76 (37.6) | 15 (51.7) | 16 (51.6) | 15 (39.5) |
| Pulmonary embolism | 32 (10.7) | 17 (8.4) | 5 (17.2) | 3 (9.7) | 7 (18.4) |
| Acute myocardial infarction | 31 (10.3) | 23 (11.4) | 4 (13.8) | 3 (9.7) | 1 (2.6) |
| Heart failure | 19 (6.3) | 10 (5.0) | 3 (10.3) | 3 (9.7) | 3 (7.9) |
| Stroke | 20 (6.7) | 14 (6.9) | 2 (6.9) | 2 (6.5) | 2 (5.3) |
| Cardiac arrest | 3 (1.0) | 2 (1.0) | 0 (0) | 1 (3.2) | 0 (0) |
| Cardiac-related ^†^ | 17 (5.7) | 10 (5.0) | 1 (3.4) | 4 (12.9) | 2 (5.3) |
| Non-cardiovascular causes | 178 (59.3) | 126 (62.4) | 14 (48.3) | 15 (48.4) | 23 (60.5) |
| Sepsis | 64 (21.3) | 44 (21.8) | 6 (20.7) | 5 (16.1) | 9 (23.7) |
| Malignancy | 67 (22.3) | 48 (23.8) | 5 (17.2) | 4 (12.9) | 10 (17.5) |
| Others | 40 (13.3) | 31 (15.3) | 1 (3.4) | 5 (16.1) | 3 (5.3) |
| Undefined | 7 (2.3) | 3 (1.5) | 2 (6.9) | 1 (3.2) | 1 (1.8) |
|  |  |  |  |  |  |
| Group 1: Normonatremia (initial serum sodium ≥135mmol/L and stayed normal during admission); Group 2: Corrected hyponatremia (initial serum sodium <135mmol/L with subsequent normalization during admission, i.e. ≥135mmol/L); Group 3: Acquired hyponatremia (initial serum sodium ≥135mmol/L, with subsequent fall during admission to <135mmol/L); Group 4: Persistent hyponatremia (initial serum sodium <135mmol/L and stayed <135mmol/L during admission). There was no statistical difference in the causes of death across the four subgroups of patients.Cardiac-related cause of death is coded when more than one cardiac cause of death is recorded on the death certificate. | | | | | |
