# Supplementary material for: Fluctuation of Serum Sodium and Its Impact on Short and Long-Term Mortality following Acute Pulmonary Embolism
Source: PLoS One. 2013 Apr 19;8(4):e61966. doi: 10.1371/journal.pone.0061966 (PMC3631139; doi:10.1371/journal.pone.0061966)
Supplement: Table S3 — Impact of simplified Pulmonary Embolism Severity Index on serum sodium predicting in-hospital all-cause mortality following acute PE.* (DOC) [file pone.0061966.s007.doc]

| **Online-only Table S3.** Impact of simplified Pulmonary Embolism Severity Index on serum sodium predicting in-hospital all-cause mortality following acute PE.* | | | | | | |
| --- | --- | --- | --- | --- | --- | --- |
| **Hyponatremia Variable** | | **Model 1** | ***p* value** | **Model 2** | ***p* value** | |
| Baseline serum sodium – per 1 mmol/L increase | | 0.89 (0.83 – 0.95) | 0.001 | 0.89 (0.83 – 0.95) | 0.001 | |
|  |  |  |  |  |  |  |
|  |  |  |  |  |  |  |
| Serum sodium change pattern | |  |  |  |  | |
| Group 1 – normonatremia versus: | | 1.00 *(reference)* | – | 1.00 *(reference)* | – | |
| Group 2 – corrected hyponatremia | | 3.62 (1.20 – 10.9) | 0.02 | 3.02 (0.97 – 9.39) | 0.20 | |
| Group 3 – acquired hyponatremia ^†^ | | – | – | – | – | |
| Group 4 – persistent hyponatremia | | 5.59 (2.08 – 15.0) | 0.001 | 6.10 (2.30 – 16.2) | <0.001 | |
| Groups 1 & 2 versus Groups 3 & 4 | | 2.17 (0.88 – 5.30) | 0.09 | 2.28 (0.93 – 5.56) | 0.07 | |
| - Unless otherwise indicated, data are presented as adjusted hazard ratio (95% confidence interval). Only univariate variables with *p*<0.10 were included in the multivariate analysis. Multivariate model 1 was adjusted for age (per 1-year), Charlson Comorbidity Index score (per 1-score) and serum hemoglobin level (per 1 g/L) on admission. Model 2 was adjusted for the simplified Pulmonary Embolism Severity Index (incorporates age, history of malignancy, cardiac failure or chronic pulmonary disease, heart rate ≥110 beats per minute, systolic blood pressure <100mmHg and arterial oxyhemoglobin <90% at admission) and serum hemoglobin level on admission. - There were no in-hospital deaths in Group 3 patients. | | | | | | |
